# Commentary: A comprehensive review of microbial contamination in the indoor environment: sources, sampling, health risks, and mitigation strategies

**DOI:** 10.3389/fpubh.2025.1654511

**Published:** 2025-11-05

**Authors:** Cesira Pasquarella, Roberto Albertini

**Affiliations:** ^1^Department of Medicine and Surgery, University of Parma, Parma, Italy; ^2^Azienda Ospedaliero-Universitaria di Parma, Parma, Italy

**Keywords:** indoor environments, air quality, bioaerosols, passive sampling, IMA standard, public health sources, sampling, health risks

Bioaerosol plays an important role in human life with potentially infectious, allergenic and toxic effects. Active and passive methods can be used to assess microbial air contamination, but so far there has been no unanimous consensus regarding the methods to be used and how to interpret the results (1–3). The active method relies on the use of volumetric samplers, enabling the measurement of the number of culturable microorganisms per unit volume of air; it measures the concentration of microorganisms in the air, and results are expressed as the number of colony-forming units (CFU) per sampling volume, usually CFU/cubic meter (m^3^). The passive method entails the use of settle plates to measure the rate at which microorganisms settle on a surface exposed to air for a defined period of time; results are expressed as CFU/plate/time. Passive sampling has been standardized by the Index of Microbial Air contamination (IMA) (4). This value corresponds to the number of CFU counted on a Petri dish 9 cm in diameter, containing nutrient agar, left open to the air according to the 1/1/1 scheme (for 1 h, 1 m above the floor and about 1 m away from walls and any major obstacles) and incubated at 37°C for 48 h. Results are expressed as IMA or as CFU/dm^2^/h.

In the Chawla's et al. (5) study, dealing with microbial contamination in the indoor environment, at point “4.1 Passive sampling” it is reported that “The air sample is collected according to the 1/1/1 scheme, plates are incubated, and results are expressed as CFU/m^3^ using the equation described by Omeliansky (63, 64). It is a simple, inexpensive, and unobtrusive sampling method (65). It gives comparable results, requires no special powered instruments or personnel, and is not influenced by engineering factors (63, 66).” The Authors referred to the scheme 1/1/1, which is the IMA standard (4), and affirmed that the results are expressed as CFU/m^3^. As previously stated, according to this standard, the results are expressed as IMA values (or CFU/dm^2^/h); therefore, it is not appropriate to express the results as CFU/m^3^ using the equation described by Omeliansky (6) as the Authors affirmed. Omeliansky proposed an equation to calculate the concentration of microorganisms (CFU/m^3^) based on the number of CFU obtained using passive sampling. The Omeliansky equation is as follows: *N* = 5*a* × 10^4^ (bt)^−1^, where: *N* = CFU/m^3^ of air, *a* = number of colonies per Petri dish, *b* = area of Petri dish in cm^2^, and *t* = exposure time in minutes.

There are several studies in which the results derived from the IMA standard are expressed as CFU/m^3^ using the equation described by Omeliansky, as reported by Viani et al. (7); however, it is important to emphasize that this is not to be considered acceptable.

Passive sampling does not measure the concentration of microorganisms in the air, expressed as CFU per volume of air, but it measures the fall-out of the biological particles, providing a measure of how the biocontamination of air contributes to the biocontamination of surfaces. This is a mirror of the airborne risk for critical surfaces (e.g. objects, material, food), and this is particularly relevant when air sampling is carried out to evaluate the risk regarding a critical surface (e.g. surgical wound, pharmaceutical product, food product, cultural artifacts) (4, 8–10). The same Authors highlighted this peculiar aspect of passive sampling when they say that “*it provides a valid risk assessment if passive sampling is performed in an operation theatre or near a surgical site*.”

It is questionable to assume that a predefined correspondence between active and passive sampling exists, as some Authors do when using specific formulae to obtain the number of CFU/m^3^ from the number of CFU/settle plates. Several studies have found a significant correlation between the results obtained by active and passive sampling, while others have failed to find such a correlation (1); however, a correlation cannot be taken for granted, since the size of the particle-carrying microorganisms is variable; there may be a case in which the concentration of microorganisms in the air is high, but the sedimentary microbial load is low, as the particles present are small in size. It is important to underscore that active and passive sampling provide distinct information, measuring different aspects of microbial air contamination. Both can be used for a general evaluation of microbial air quality; however, according to the aim of microbial sampling, the choice can fall on one or the other. For example, if the interest is to assess the exposure through inhalation, active sampling is preferable, while if the interest is to evaluate the risk of contamination for a critical surface, passive sampling can give a more relevant answer.

As for the Omeliansky equation (6), which was published in 1940, it is notable that CFU/m^3^ values calculated by applying the Omeliansky equation to the values from passive sampling, are much higher than the values which would be derived from the relationship between active and passive sampling provided by the EC GGMP Guidelines to Good Manufacturing Practice (11) or found in a multicentric study in conventionally ventilated operating theaters (12). For example, a value of 25 CFU counted on a Petri dish 9 cm in diameter left open for 1 h corresponds to 327 CFU/m^3^ applying the Omeliansky equation and to 200 CFU/m^3^ applying the EC GCMP or the equation from the study by Pasquarella et al. When using the standard IMA, the value must not be converted into CFU/m^3^; for an estimate of the CFU/m^3^, EC GGMP relationship or the equation by Pasquarella et al. (12) could be used.

In a context in which there are no generally accepted protocols for the assessment of microbial air contamination, it is important to have appropriate and clear knowledge of the available sampling methods to be used, and the review by Chawla et al., provides a comprehensive overview of microbial contamination in built environments, covering sources, sampling strategies, and analysis methods. However, we ask for a revision of the use of the Omeliansky equation for expressing the results after collecting an air sample by passive sampling, according to the 1/1/1 scheme. This would avoid misunderstandings and inaccurate communication.
